# Clinical Characteristics and Variation in Musculoskeletal Complexity of Different Ethnic Populations Accessing Sandwell and West Birmingham Hospital's MSK Service: A Service Evaluation

**DOI:** 10.1002/msc.70012

**Published:** 2024-11-24

**Authors:** Wasim Shah, Roger Newham, Carolyn Casey, Roanna Burgess

**Affiliations:** ^1^ Musculoskeletal Service Sandwell and West Birmingham NHS Hospitals Birmingham UK; ^2^ Institute of Clinical Sciences University of Birmingham Birmingham UK

**Keywords:** clinical characteristics, ethnicity, health inequalities, musculoskeletal

## Abstract

**Introduction:**

Health inequality is a global public health challenge, limited by insufficient high‐quality data and analysis. Musculoskeletal (MSK) pain disorders are more prevalent among ethnic minority groups disproportionately affected by socioeconomic disparities and poor health outcomes. Ethnicity data collection enables NHS organisations and policymakers to understand specific healthcare needs and ensure equitable access and care provision.

**Objective:**

To understand the baseline clinical characteristics across ethnic population groups accessing MSK care at Sandwell and West Birmingham (SWB) NHS Trust.

**Methods:**

Retrospective analysis of routine data collected using patient self‐report surveys (August 2020–February 2023). Core metrics included demographics, pain characteristics, and Patient Reported Outcome Measures (PROMs) including the Musculoskeletal Health Questionnaire (MSK‐HQ) and Numeric Pain Rating Scale (NPRS). Descriptive statistics and statistical tests were undertaken, with means, percentage values and variation by ethnic groups across baseline MSK‐HQ and pain scores reported.

**Results:**

Survey data were provided by 13,248 patients, with 7295 (55.06%) stating their ethnicity. Statistical differences were found between baseline MSK‐HQ and NPRS scores between ethnic groups. The mean MSK‐HQ score was 24.1 overall, lowest in the Asian group (22.3) and highest in the Mixed group (24.8). Mean baseline pain intensity was (7.8), highest in the Asian group (8.3). One or more comorbidities were present in 46% of patients, with the highest percentage in the White and Black groups sequentially.

**Conclusion:**

Baseline health inequalities exist among ethnic groups accessing the SWB MSK service. Disparities may be associated with patient or system barriers and require further exploration.

## 1 Background

### 1.1 Introduction

The demands on health services worldwide are increasingly challenged by escalating changes in population demographics and evolving healthcare needs (Evans et al. 2020). It is recognised that health inequality is a global public health challenge and a moral imperative concerning social justice (Regmi and Mudyarabikwa 2020). Understanding of ethnic health inequalities continues to be limited by a lack of health service‐specific data collection and analysis (Kapadia et al. 2022). Ethnic health inequality is associated with the variation in access to opportunities and resources for an individual or group identifying as being from an ethnic background (Hills et al. 2010). The origins of ethnic health disparities are difficult to identify, but research suggests a complex interaction between deprivation and environmental, cultural and physiological health related behaviours between generations (Raleigh and Holmes 2021). People from Black and Minority Ethnic (BAME) backgrounds are disproportionately affected by socioeconomic deprivation, are less likely to access specialist services, have poorer experiences of primary care, and report worse outcomes when compared to their White counterparts (Livingston et al. 2002; Watkinson, Sutton, and Turner 2021; Peters et al. 2009). The COVID‐19 pandemic exacerbated pre‐existing health disparities, emphasising the need for policy makers to address diverse health needs and improve healthcare management in underprivileged and BAME communities (Robertson et al. 2021; Raleigh and Holmes 2021).

The 2017, Health Survey for England reported that 5.5 million people were affected by moderately to severely disabling chronic pain, which was not experienced equally across population groups (Public Health England 2017a, 2017b; Jeraj and Butt 2020; Allison et al. 2002). Persistent MSK pain places a vast personal and economic burden on the sufferer, affects around 30% people worldwide and is likely to increase given the ageing population (Cohen, Vase, and Hooten 2021). MSK pain is multi‐dimensional and is associated with a higher prevalence of risk factors including physical inactivity, vitamin D deficiency, socioeconomic deprivation and other pre‐existing health conditions common in BAME communities (Allison et al. 2002) such as diabetes and cardiovascular disease (Raleigh and Holmes 2021). Watson, Latif, and Rowbotham (2005) found an ethnic variation was present, with British South Asians having a lower pain threshold and increased pain intensity compared with their White British counterparts.

The Five Year Forward View (NHS England 2014) established unwarranted variation in patient care and outcomes as a priority for NHS transformation, but higher quality data are required to understand the health differences between ethnic minority groups. Individuals who identify as being from BAME backgrounds may recover more slowly from MSK health issues without appropriate care when compared to their White counterparts and suffer longer term ill effects (Marmot et al. 2020).

Ethnic health inequalities exist where there are higher levels of migration—the movement from the country of birth to a country of residence (Smyth 2008; Barbek et al. 2022). Migration is particularly high and growing in the West Midlands, accompanied by worsening general health, significantly lower life expectancy and increasing levels of deprivation (Public Health England 2017a, 2017b). Gaining timely access to physiotherapy services has been a long‐standing problem which particularly affects more socioeconomically deprived areas (CSP 2011). There is a national drive to increase focus on the most deprived communities, where engagement with health services is lower than expected given the incidence of MSK conditions (Arthritis and Musculoskeletal Alliance (ARMA) 2021).

Under the Race Relations (Amendment) Act (2000), public health authorities are legally obliged to promote equal opportunities and monitor outcomes by ethnicity, but a King's Fund review of 300 Primary Care Trusts found that a third failed to comply (Ross et al. 2020). The NHS struggles to maintain standards of care in the face of operational issues and financial pressures (Alderwick et al. 2017) and the coverage and quality of data examining ethnic health differences is substandard (Raleigh and Holmes 2021). The King's Fund (2017) recommends that NHS organisations should prioritise improving the quality of care among BAME groups. Collecting ethnicity data is therefore crucial for health care professionals, NHS organisations and policymakers to better understand the disparities faced by ethnic groups and enable design of health services tailored to population need (NHS Digital 2022). This evaluation focused on the borough of Sandwell, ranked the 12th most deprived local authority in the West Midlands, where 48% residents identify as being from BAME communities (Census 2021).

### 1.2 Aim

To describe the baseline clinical characteristics across ethnic population groups accessing Sandwell and West Birmingham (SWB) MSK service from August 2020 to February 2023 in order to understand the potential variation in health severity of patients of different ethnic groups at the initial point of access.

### 1.3 Objectives

- To group patients by reported ethnicity and describe demographics and clinical characteristics to include age, gender, referral source, presence of comorbidities and Patient Reported Outcome Measures (PROM) including Numeric Pain Rating Scale (NPRS) and Musculoskeletal Health Questionnaire (MSK‐HQ) score at baseline (prior to treatment).
- To respond to data analysis by proposing quality improvement (QI) activity targeting unwarranted variation between patient groups accessing care and comply with Race Relations legislation.

## 2 Methods

### 2.1 Study Design

The Standards for Quality Improvement Reporting Excellence 2.0 (SQUIRE) guidelines were used as a framework for systematically reporting this service evaluation. SQUIRE is recommended to describe system level work to improve the quality, safety and efficiency of healthcare (Ogrinc et al. 2015).

### 2.2 Setting and Participants

This project was conducted within an integrated NHS care organisation located in the West Midlands, United Kingdom. The Trust is responsible for the care of 530,000 local people and approximately 520,000 people attend consultations annually in outpatient departments, including MSK services (NHS England, 2019 (Samuda and Lewis 2019)). This evaluation retrospectively analysed data for patients ≥ 16 years old who had attended for MSK care and who had completed a routine patient survey on their clinical symptoms and health status at the start of their care episode.

### 2.3 Data Collection

A retrospective evaluation of routine MSK patient survey data was conducted. Baseline survey data were available for 13,248 patients seen for the first time between August 2020 and February 2023. This project examined and reported on baseline data for 7295 patients with a stated ethnicity seen during the specified time period. The SWB MSK Service uses a standardised set of core metrics on descriptive characteristics of participants, pain‐related characteristics and PROMs, based on a previous study by Burgess et al. (2021). The dataset comprised demographics (age, ethnicity, gender), source of referral (primary, secondary, tertiary, self), clinical descriptors (average number of comorbidities), and PROMs (Numeric Pain Rating Scale [NRPS], Musculoskeletal Health Questionnaire [MSK‐HQ]) collected upon initial presentation to the MSK service. The MSK‐HQ is scored out of 56, with a higher score representing a better MSK health status (Westby et al. 2014). The NPRS is a unidimensional measure of pain intensity scored out of 10 (Hill et al. 2016). The scale is composed of 0 (no pain at all) to 10 (worst imaginable pain). Source of referral was a key measure to identify equal access opportunities. Comorbidity refers to the presence of two or more medical conditions in the same individual. A summary of patient reported data collected at baseline can be found in Supporting Information S1: Table S1 and referral source information can be found in Supporting Information S1: Table S7.

The data collection software used was CoMetrica, a third‐party company commissioned by SWB NHS Trust, which uses an electronic system to capture patient survey data. An electronic data collection process demonstrates data integrity and is more efficient than paper‐based data collection methods with increasing data accuracy, timeliness and cost‐effectiveness (Mosa, Yoo, and Parker 2015). Upon booking an initial MSK consultation, the administration team check contact email and phone details and informs the patient to expect an email from CoMetrica inviting them to complete the patient survey. A data sharing process was initiated to allow data transfer between the patient, the electronic patient record and CoMetrica. A Data Protection Impact Assessment (DPIA) was completed with the Trust Governance Team prior to commencement. A privacy statement was also available to advise patients about the safety of their data prior to completion. The developed dataset was built within CoMetrica to capture data at the start and towards the end of an episode of MSK care.

### 2.4 Data Analysis

Data analysis was conducted using Microsoft Office software (Microsoft 2023). An excel spreadsheet was generated by CoMetrica with the dataset provided in a statistically coded format. For the analyses of this project, ethnicity was grouped into categories of Asian/Asian British, Black British/African/Caribbean, White, Mixed and Other ethnic groups, to draw comparisons between groups. Where there were data without an ethnic group listed, ethnicity was reported as Blank/Missing. Pivot tables were created in Microsoft Excel. Tables were used to illustrate the findings. Descriptive statistics were used to describe measures of central tendency (mean or median), percentage values and measures of variability (spread) to summarise data (Manikandan 2011).

Further statistical analysis of the MSK‐HQ and pain data was conducted in SPSS. The variances and group sizes in ethnic groups for MSK‐HQ and pain were unequal; therefore, a comparison of means was conducted using Welch ANOVA and a Games‐Howell post hoc test (Field 2018).

The alpha level denoting significance was set at 5%.

## 3 Results

### 3.1 Baseline Characteristics

A total of 28,434 new patients were sent a questionnaire between August 2020 and February 2023, which was completed by 13,248 (46.59%) patients. Ethnicity was available for 7295 patients (55.06%) and was missing in 44.94% of the total responses. For the purpose of this analysis, the sample was 13,248 patients with baseline data, with a sub‐analysis of those with a stated ethnicity.

An overview of descriptive data for baseline socio‐demographic and clinical characteristics with ethnicity breakdown is presented in Supporting Information S1: Table S2.

Baseline PROM (NPRS), MSK‐HQ, referral sources and patient characteristics including gender and comorbidities are tabulated according to ethnic groups. The largest group of respondents described their ethnicity as White (*n* = 4043 [30.52%]), followed by Asian (*n* = 1689 [12.75%]), Black (*n* = 601 [4.54%]), Mixed (*n* = 526 [3.97%]) and Other ethnicity (*n* = 436 [3.29%]). This follows similar ethnicity population trends as per the 2021 Census for the Sandwell area, which reports 57% of the population as White and 43% representing the non‐white minorities (Census 2021).

There were 13,248 respondents with a baseline patient reported MSK‐HQ. The mean MSK‐HQ was 24.1 for the total population (see Supporting Information S1: Table S2).

The age group most commonly attending the service was 50 to 59‐year‐olds, accounting for 22.62% of all patients. This was the most common age band observed across all groups. The Black, Asian and Mixed ethnic minority groups were younger on average in their median age than the White and Other ethnic minority groups.

There were considerably more female (*n* = 7885 [61.65%]) than male (*n* = 4903 [38.34%]) respondents across all groups, with the average MSK‐HQ score for males (25.1) being higher (better MSK health status) than females (23.5) overall. Asian women had the lowest MSK‐HQ score of 21.3 out of all the female and male group categories. Black and Mixed males had the highest MSK‐HQ score (26.0) of all ethnicity groups (male and female). Of the male groups, Other males had the lowest mean MSK‐HQ score of 23.8.

Comorbidity was present in 46% of the total population who reported 1 or more comorbidities. The Asian population had the highest percentage of 0 reported comorbidities [*n* = 1031 (60.75%)], whereas the White population group had the highest percentage of 3 or comorbidities [*n* = 496 (12.2%)] of all ethnic groups. Within the category ‘3 or more comorbidities’, the Asian population group had the lowest percentage [*n* = 113 (6.65%)]. Supporting Information S1: Table S2 illustrates that comorbidities are not evenly spread across ethnic groups.

Of the 7295 patients who reported ethnicity, the lowest MSK‐HQ score was seen in the Asian group, who reported a score of 22.3 (see Supporting Information S1: Table S3). The highest MSK‐HQ score at baseline (24.8) was reported by the Mixed group, followed by the White, Other and Black ethnic groups sequentially.

Post hoc testing identified significant differences in the mean MSK‐HQ total score in some but not all comparisons (see Supporting Information S1: Table S4).

The average baseline pain intensity score on the NPRS for the total population was 7.8 (see Supporting Information S1: Table S2). Of the 7295 who reported ethnicity, the mean pain was 7.95 (SD 2.16) (see Supporting Information S1: Table S5). Pain intensity was greatest in the Asian group, with an average score of 8.3. The scores were followed by Black and Other groups sequentially. Pain intensity was lower for the White and Mixed groups (mean NPRS 7.8).

There was a significant difference in pain scores between the different ethnic groups (*F*_4_ = 16.231, *p* < 0.001) (see Supporting Information S1: Table S6).

Within group differences show a slightly higher pain score in Black/Black British and Asian/Asian British participants in comparison to those who are White, Mixed or Other (Supporting Information S1: Table S6).

Supporting Information S1: Table S7 shows the total referrals received during the 30‐month reporting period (28,434). Referrals were split into their corresponding referral sources (self‐referrals, primary care, secondary care, community and other). Most referrals were from Primary care [*n* = 11,694 (41.13%)], followed by Secondary care [*n* = 9058 (31.86%)], self‐referral [*n* = 6624 (23.3%)], other [*n* = 1033 (3.63%)] and community [*n* = 25 (0.09%)]. The White group had the lowest percentage of self‐referral of all groups, but the highest percentage of referrals from primary care. All other referral sources appeared to be equitable across ethnic groups.

## 4 Discussion

To the author's knowledge, this is the first service evaluation reporting key baseline characteristics within ethnic groups accessing MSK care in the UK. Comparison to previous literature is therefore not possible, but reference to similar work is attempted. This evaluation has highlighted potential unwarranted variation between ethnic groups and health outcome data. Self‐reported patient outcome data is increasingly used in clinical settings to capture the clinical characteristics of patients and to assess the impact of various interventions (Wade et al. 2011). The low baseline MSK‐HQ score for the total population (24.1) infers complexity within the diverse communities accessing SWB MSK services, together with the challenge in providing personalised care. In contrast, the national First Contact Practitioner (FCP) service evaluation reported a significantly higher mean MSK‐HQ score of 33.8 (Stynes et al. 2021), suggesting that the MSK health status of the local population is worse than the national average. It is acknowledged that the FCP evaluation was undertaken within primary care rather than a community setting and that the ethnic representation within the evaluation was low, with 97% respondents in the White ethnic category, making comparisons difficult. Further support is provided by a recent publication highlighting the increase in risk factors for ill health in the health profile of patients as a whole, living in the West Midlands (Public Health England 2021). This lack of national data representative of wider ethnic groups can perpetuate health disparities, as it does not capture the unique health needs of specific ethnic communities. Notably, this local service evaluation shows that MSK‐HQ scores were consistently low for all groups; however, they are worse for those from BAME backgrounds. This mirrors previous literature that worsening socioeconomic drivers exacerbate health inequalities in the most socially deprived areas (Public Health England 2021). Patients from BAME communities are more likely to experience poor social determinants of health, for example, because of living in neighbourhoods with poor air quality, limited access to healthy food options and higher rates of violence, that negatively impact their health status. This can result in differences in the way geographically deprived BAME groups report their outcomes, as they may have different priorities and concerns than patients from more advantaged backgrounds (Raleigh and Holmes 2021).

Supporting Information S1: Table S4 shows for example, that the Asian group had significantly lower MSK‐HQ scores compared to the Mixed group (mean difference *−*2.495, *p* < 0.001), indicating a notable disparity in how these groups experience or report their musculoskeletal health. In particular, Asian females presented with the lowest MSK‐HQ scores. A systematic review by Rahim‐Williams et al. (2012) found that ethnic differences in pain perception may be influenced by sociocultural, psychological and biological mechanisms. Sociocultural factors influencing pain sensitivity could include beliefs and attitudes of a group's ethnic identity, which may predict coping style through locus of control (Bates, Edwards, and Anderson 1993). Individuals who more strongly identify with their ethnic group, due to the knowledge, value and emotional significance they attach to that culture, may be more sensitive to pain. Psychological factors associated with differences in ethnic group pain responses may include higher levels of catastrophising and passive coping as reported in BAME groups associated with greater pain sensitivity (Rahim‐Williams et al. 2012). From a biological viewpoint, differences in pain perception between ethnic groups may be due to alterations in endogenous pain control mechanisms. Kim et al. (2004) found that allele frequencies for single nucleotide polymorphisms (SNP) of pain genes differed between ethnic groups, suggesting that genetic factors may contribute. The statistically significant differences in MSK‐HQ scores between ethnic groups may also be a reflection of psychological factors because groups with higher levels of passive coping may perceive less pain and report lower scores. Disparities between ethnic groups are non‐uniform, indicating a need for personalised approaches to address health inequalities across different communities.

Baseline differences may also be influenced by patient and system barriers. Patient barriers refer to individual‐level factors that prevent patients from accessing healthcare (Yin et al. 2020), such as low health literacy, cultural health beliefs/behaviours and transportation barriers. Patients from ethnic minority groups may experience difficulties with access due to language barriers and cultural beliefs together with social and economic disadvantage. System barriers refer to healthcare structures and practices that prevent equitable access and outcomes (Yin et al. 2020). These can include factors such as lack of cultural competence amongst staff, treatment criteria, inadequate interpreting services, waiting lists and limited availability of healthcare services in certain areas. System barriers can prevent patients from accessing healthcare or result in suboptimal care, which can lead to health disparities between different ethnic groups. A systematic review by Ocloo et al. (2021) supports this concept and provides valuable insights into the barriers faced by patients and the public in accessing healthcare services in the UK. Key barriers for patients were related to language proficiency and the need to feel empowered to deal with their health condition. Other barriers were described at organisational level with patient and professional engagements needing to be conducted in a non‐paternalistic manner to ensure people do not feel subordinate to clinicians. However, the review only included studies that were published in English, which may have excluded relevant studies published in other languages, limiting comprehensiveness and introducing bias. Also, the review primarily focused on barriers faced by patients and the public in accessing healthcare services but did not extensively explore the interaction between healthcare professionals and patients as a potential contributing factor.

Pain intensity was high for all groups, as the mean score for the total population was 7.8, which is higher than a national evaluation which reported a mean pain intensity of 6.1 (Stynes et al. 2021). Stevenson et al. (2024) reported mean baseline pain intensity scores of 7.2 for the MIDAS study control GP cohort presenting with MSK pain within practices in Staffordshire, and 5.79 for the lower risk SelfSTarT app intervention users. These differences suggest that the SWB population may experience musculoskeletal symptoms of greater severity compared with other local and national population groups. Variations in patient demographics, the types of conditions treated, sample size and recruitment methodologies may account for these discrepancies, highlighting the need to consider local patient characteristics when interpreting pain intensity scores. Furthermore, comorbidity was present in 46% of the total population. This highlights the complexity of the local patient population and implies a greater disease burden. Pain intensity score was greatest for the Asian group as a whole, followed by the Black and Other groups, respectively. Interestingly, the Black group had a high pain intensity score and reported a high percentage of three or more comorbidities. This suggests a relationship between pain and comorbidities; individuals from BAME groups and lower socioeconomic backgrounds may be at increased risk for chronic pain and associated comorbidities (Raleigh and Holmes 2021). The statistical findings in Supporting Information S1: Table S6 show significantly higher pain scores in the Asian or Asian/British individuals compared to White participants (mean difference −0.484, *p* < 0.001). This highlights a notable disparity in pain experience within this ethnic group. Additionally, the Black/Black British group also showed slightly higher pain scores compared to White participants (mean difference −0.324), though this was not statistically significant (*p* = 0.097). These findings suggest that Asian and Black populations report higher baseline pain than White individuals, which may be driven by both socioeconomic factors and disparities in access to pain management strategies. The Mixed group, however, reported significantly lower pain scores compared to Asian or Asian British individuals (mean difference 0.465, *p* < 0.001), highlighting some variability in pain experience even within BAME groups.

Variations at baseline between groups may also be explained by differences in language and communication. Differences in language, sub‐optimal access to translation services and health literacy may affect how patients understand and complete PROMs. Language barriers may lead to misunderstandings and inaccuracies in PROMs, while low health literacy may lead to difficulties in understanding and responding to the questionnaires in a way that accurately reflects an individual's health status. An additional point to consider is digital literacy, as the patient survey was completed online. This was similar to the national FCP evaluation, which also invited patients to complete a survey by email with a response rate of 24% (Stynes et al. 2021). The response rate in this real‐world service evaluation was considerably higher (46.59%) but may still have been influenced by several patient factors such as, reduced skills in health literacy to complete the survey, or digital poverty. Health Education England (2016) funded an online health literacy geodata tool which provides an estimate of the percentage of a local authority population with low health literacy and numeracy and found that in the South region of the country, approximately 43% of the population was below the average for health literacy. Although the tool has not been developed in other regions, it highlights challenges whereby population groups nationally are unable to understand health information sufficiently well to make informed health decisions. The difficulties patients may have in communicating their symptoms and concerns to healthcare providers can lead to misunderstandings, misdiagnoses and inadequate treatments, further impacting the accuracy of the use of tools such as PROMs.

This service evaluation found that primary care referrals were disproportionately high for the White group. In contrast, self‐referrals were predominantly from Black and Asian populations. This disparity suggests the presence of system barriers to access within primary care, particularly for non‐White ethnic groups. These barriers may stem from referrer bias leading to reduced referral rates for minority ethnic groups, which could contribute towards the differences in baseline PROM scores. However, the data does not clarify whether these groups face linguistic challenges or require support from family members when navigating self‐referral pathways, particularly for non‐English speaking patients. Research suggests that ethnic groups face barriers to accessing healthcare services due to discrimination and systemic inequalities (Hall et al. 2015). This leads to delayed diagnosis and treatment, which negatively impacts an individual's health quality status and outcomes. A systematic review by Hall et al. (2015) found that ethnic minorities often face discrimination and bias from healthcare providers. A study from the US investigated the association between clinicians' beliefs and racial disparity in pain management between ethnic groups and found that clinicians' medical judgement may be influenced by beliefs surrounding biological differences between Black and White patients, which may contribute to unintentional ethnic inequalities (Hoffman et al. 2016). For example, primary care practitioners may assume that certain symptoms are more or less significant depending on a patient's ethnicity, or that certain ethnic groups are less likely to comply with treatment plans. These biases may result in delays in referrals to services such as MSK Physiotherapy, leading to inequalities in healthcare delivery.

### 4.1 Implications

This service evaluation is beneficial to local stakeholders by providing improved understanding of the health inequalities across the local population. This project provides a potential model for future national evaluation focused on understanding and improving the root causes of health disparities between different ethnic groups supporting the Arthritis and Musculoskeletal Alliance's (ARMA) national inquiry into inequalities in MSK health (ARMA 2024).

Healthcare plays a contributory role in addressing inequalities. Good quality data is essential for enabling policy makers and local services to identify and control for levels of need within different ethnic groups (The King's Fund, 2017). Further evaluation will be conducted on pre and post‐treatment scores to identify whether changes in PROM scores following MSK physiotherapy intervention are statistically significant and whether post‐treatment outcomes are reflected equally between the ethnic groups. Qualitative research to understand patients' experiences when accessing MSK care and their perceptions of barriers and delays would also improve understanding of issues faced and enable themes and recommendations for quality improvement to be generated. There is a lack of guidance on using outcome data to directly improve patient outcomes, experiences and value (Burgess et al. 2021), together with scarcity in the development of theory‐driven approaches to improving understanding at an individual, organisational and socio‐political level. Further research is therefore needed to explore the reasons for variations in patient‐reported outcome data and identify optimal MSK care pathways.

### 4.2 Limitations

The data collection time frame partly overlapped the COVID‐19 pandemic and may not therefore be indicative of normal service activity levels, but the time frame utilised allowed a large sample size to be adopted. There may also be bias within the sample as only those with a certain level of digital literacy are likely to have participated. Ethnicity was missing in 40.75% of cases, which may have introduced bias. The MSK service has moved to self‐reporting ethnicity to support further work exploring this. It is important to consider and address the reasons for missing data; hence, there is also a need for qualitative data collection to further develop understanding of the population.

## 5 Conclusion

In conclusion, this project meets its aims of reporting on ethnicity related health data to increase understanding of the differing needs of SWB's diverse population. It has been identified that baseline health inequalities exist between ethnic groups. A variety of factors that contribute to baseline differences in PROMs between ethnic groups have been discussed, including social and economic factors, cultural differences in health beliefs, language and communication barriers, health provider discrimination and bias. Although many of these factors are non‐modifiable, health professionals and organisations should be aware of them and their impact on a patient's ability to access appropriate and timely care. Research associated with ethnic health inequities and MSK conditions is limited and the associations discussed merit further investigation locally and at the national level to create a more inclusive and culturally responsive healthcare system that provides equitable access to healthcare services for all.

## Author Contributions

**Wasim Shah:** conceptualization (lead), data curation (lead), formal analysis (lead), methodology (lead), writing–original draft preparation (lead), writing–review and editing (lead). **Roger Newham:** supervision (lead), validation (supporting), writing–review and editing (supporting), formal analysis (supporting). **Carolyn Casey:** conceptualization (supporting), formal analysis (supporting), methodology (supporting), validation (supporting), writing–review and editing (supporting), supervision (supporting). **Roanna Burgess:** conceptualization (supporting), methodology (supporting), validation (supporting), writing–review and editing (supporting).

## Ethics Statement

This project was deemed to be a service evaluation. Ethical approval was therefore not required.

## Conflicts of Interest Statement

The authors declare no conflicts of interest.

## Supporting information

Supporting Information S1

## Data Availability

The data that support the findings of this study are available on request from the corresponding author. The data are not publicly available due to privacy or ethical restrictions.
