# Supplementary material for: Clinical Characteristics and Variation in Musculoskeletal Complexity of Different Ethnic Populations Accessing Sandwell and West Birmingham Hospital's MSK Service: A Service Evaluation
Source: Musculoskeletal Care. 2024 Nov 24;22(4):e70012. doi: 10.1002/msc.70012 (PMC11586323; doi:10.1002/msc.70012)
Supplement: Supplementary file 1 — Supporting Information S1 [file MSC-22-e70012-s001.docx]

Supplementary file with Tables

**Table 1** Patient Descriptors

| **Patient Descriptors** | **Definition** | **Source of data collection** |
| --- | --- | --- |
| Age | Age at initial consultation | Electronic health record |
| Ethnicity | What is your ethnicity? (Mixed, Asian, Black, White, Other) | Electronic health record |
| Comorbidities | Self-reported diagnosed comorbidities from a provided list: heart disease, high blood pressure, poor circulation, lung disease, diabetes, kidney disease, neurological disorder, liver disease, cancer, depression, arthritis | Baseline patient survey |
| Gender | Sex at birth | Electronic health record |
| MSK-Health Questionnaire (MSK-HQ) | Impact from MSK symptoms (15 questions)  The MSK-HQ is scored out of 56, with a higher score representing a better MSK health quality, the higher the score. | Baseline patient survey |
| Numeric Pain Rating Scale (NPRS) | Average pain intensity during the last 2 weeks NPRS (0-10)  The NPRS is scored out of 10, with a lower score representing lower pain intensity. | Baseline patient survey |

**Table 2** Descriptive statistics summary table

| **All patients** | **Total**  N= 13248 | Mixed  N= 526 | Asian  N= 1689 | Black  N= 601 | White  N= 4043 | Other  N= 436 | Blank  N= 5953 |
| --- | --- | --- | --- | --- | --- | --- | --- |
| **MSK-HQ baseline score**  **mean and (SD)** | 24.1 (10.53) | 24.8 (10.49) | 22.3 (9.86) | 23.0 (10.49) | 24.6 (10.34) | 23.3 (10.84) | 24.4 (10.76) |
| P**ain intensity baseline score**  **Mean and (SD)** | 7.8 (2.23) | 7.8 (2.20) | 8.3 (2.06) | 8.1 (2.15) | 7.8 (2.18) | 7.9 (2.21) | 7.7 (2.32) |
| **Age: median** | 55 | 50 | 50 | 44 | 55 | 55 | 55 |
| **Age- band: n (%)**  10-19  20-29  30-39  40-49  50-59  60-69  70-79  80-89  90-99  100-109 | 322 (2.39%)  1343 (9.96%)  2093 (15.52%)  2456 (18.21%)  3051 (22.62%)  2184 (16.19%)  1012 (7.5%)  994 (7.37%)  33 (0.24%)  1 (0.01%) | 20 (3.8%)  62 (11.79%)  73 (13.88%)  100 (19.01%)  131 (24.9%)  83 (15.78%)  48 (9.13%)  7 (1.33%)  1 (0.19%)  1 (0.19%) | 46 (2.72%)  163 (9.65%)  336 (19.89%)  407 (24.09%)  361 (21.37%)  246 (14.56%)  96 (5.68%)  34 (2.01%)  -  - | 22 (3.66%)  58 (9.65%)  90 (14.97%)  122 (20.29%)  169 (28.11%)  110 (18.30%)  17 (2.82%)  13 (2.16%)  -  - | 99 (2.44%)  289 (7.14%)  487 (12.04%)  620 (15.33%)  1039 (25.69%)  838 (20.72%)  501 (12.39%)  151 (3.73%)  19 (0.46%)  - | 9 (2.06%)  55 (12.61%)  86 (19.72%)  124 (28.44%)  82 (18.80%)  47 (10.77%)  20 (4.58%)  9 (2.06%)  4 (0.91%)  - | 126 (2.29%)  716 (13.02%)  1021 (18.58%)  1083 (19.70%)  1269 (23.09%)  860 (15.65%)  331 (6.02%)  80 (1.45%)  9 (0.16%)  - |
| **Gender: n (%)**  Male  Female | 4903 (38.34%)  7885 (61.65%) | 194 (36.88%)  332 (63.11%) | 606 (35.87%)  1083 (64.12%) | 165 (27.45%)  436 (72.54%) | 1390 (34.38%)  2652 (65.61%) | 147 (33.71%)  289 (66.28%) | 2401 (43.70%)  3093 (56.29%) |
| **MSK-HQ baseline score by Gender (mean)**  Male  Female | 25.1  23.5 | 26.0  24.2 | 24.3  21.3 | 26.0  21.9 | 25.8  24.0 | 23.8  23.1 | 24.9  23.9 |
| **NRPS baseline score by Gender (mean)**  Male  Female | 7.6  8.0 | 7.5  8.0 | 8.0  8.5 | 7.6  8.3 | 7.5  8.0 | 8.0  7.8 | 7.5  7.8 |
| **Comorbidity Count: n (%)**  0  1  2  3+ | 7181 (54%)  3418 (25.7%)  1481 (11.1%)  1219 (9.2%) | 261 (49.4%)  152 (28.73%)  72 (13.61%)  44 (8.32%) | 1031 (60.75%)  385 (22.68%)  168 (9.89%)  113 (6.65%) | 309 (50.90%)  158 (26.02%)  71 (11.70%)  69 (11.36%) | 1876 (46.22%)  1094 (26.96%)  592 (14.59%)  496 (12.22%) | 265 (60.50%)  102 (23.29%)  38 (8.68%)  33 (7.53%) | 3439 (57.60%)  1527 (25.57%)  540 (9.05%)  464 (7.77%) |

**Table 3** Mean MSK-HQ for each ethnic group

| Ethnicity | N | Mean MSK-HQ | SD | 95% CI for Mean |
| --- | --- | --- | --- | --- |
| White | 4043 | 24.6 | 10.34 | 24.28 to 24.91 |
| Black or Black British | 601 | 23.04 | 10.50 | 22.20 to 23.88 |
| Asian or Asian British | 1689 | 22.34 | 9.86 | 21.86 to 22.81 |
| Other | 436 | 23.34 | 10.84 | 22.32 to 25.73 |
| Mixed | 526 | 24.83 | 10.50 | 23.93 to 25.75 |
| Total | 7295 | 23.89 | 10.33 | 23.65 to 24.12 |

**Table 4** Significant (at p<0.05) differences between ethnic groups for total MSK-HQ.

| Group 1 | Group 2 | Mean difference | SE | P | 95% confidence interval |
| --- | --- | --- | --- | --- | --- |
| White | Black or Black British  Asian or Asian British | 1.552  2.260 | 0.007  0.290 | 0.007  <0.001 | 0.3 to 2.80  1.47 to 3.05 |
| Asian or Asian British | Mixed | -2.495 | 0.517 | <0.001 | -3.91 to -1.08 |
| Black or Black British | Mixed | -1.788 | 0.627 | 0.036 | -3.50 to -0.08 |
| Other | White | -1.254 | 0.544 | 0.145 | -2.74 to 0.23 |

**Table 5** Mean pain scores by ethnicity

| Ethnicity | N | Mean NPRS | SD | 95% CI for Mean |
| --- | --- | --- | --- | --- |
| White | 4043 | 7.81 | 2.18 | 7.74 to 7.88 |
| Black or Black British | 601 | 8.14 | 2.15 | 7.96 to 8.32 |
| Asian or Asian British | 1689 | 8.30 | 2.06 | 8.19 to 8.40 |
| Mixed | 526 | 7.83 | 2.20 | 7.64 to 8.03 |
| Other | 436 | 7.87 | 2.21 | 7.65 to 8.09 |
| Total | 7295 | 7.95 | 2.16 | 7.90 to 8.01 |

**Table 6** Differences in baseline pain scores across ethnic groups

| Group 1 | Group 2 | Mean difference | SE | p | 95% confidence interval |
| --- | --- | --- | --- | --- | --- |
| White | Black or Black British  Asian or Asian British | -0.324  -0.484 | 0.097  0.063 | 0.008  <0.001 | -0.59 to -0.06  -0.66 to -.031 |
| Asian or Asian British | Other  Mixed | 0.425  0.465 | 0.123  0.111 | 0.006  <0.001 | 0.09 to 0.76  0.16 to 0.77 |

**Table 7** Source of referral

| **All patients** | **Total**  N= 28434 | Mixed  N= 2817 | Asian  N= 6061 | Black  N= 2503 | White  N= 6851 | Other  N= 2590 | Blank  N= 7612 |
| --- | --- | --- | --- | --- | --- | --- | --- |
| **Source of referral: n (%)**  Self-referral  Primary care  Secondary care  Community  Other | 6624 (23.3%)  11694 (41.13%)  9058 (31.86%)  25 (0.09%)  1033 (3.63%) | 720 (25.55%)  1151 (40.86%)  831 (29.5%)  2 (0.07%)  113 (4.01%) | 2045 (33.74%)  2332 (38.48%)  1555 (25.66%)  10 (0.16%)  119 (1.96%) | 895 (35.76%)  853 (34.08%)  663 (26.49%)  4 (0.16%)  88 (3.52%) | 1110 (16.2%)  4090 (59.7%)  1449 (21.15%)  4 (0.06%)  198 (2.89%) | 743 (28.69%)  1027 (39.65%)  744 (28.73%)  -  76 (2.93%) | 1111 (14.6%)  2241 (29.44%)  3816 (50.13%)  5 (0.66%)  439 (5.77%) |
